# Poly-protein G-expressing bacteria enhance the sensitivity of immunoassays

**DOI:** 10.1038/s41598-017-01022-w

**Published:** 2017-04-20

**Authors:** Wen-Rui Hao, Michael Chen, Yi-Jou Chen, Yu-Cheng Su, Chiu-Min Cheng, Hsiang-Yin Hsueh, An-Pei Kao, Yuan-Chin Hsieh, Johny Chang, Ming-Yang Tseng, Kuo-Hsiang Chuang

**Affiliations:** 1 Division of Cardiovascular Medicine, Department of Internal Medicine, Shuang Ho Hospital, Taipei Medical University, New Taipei City, Taiwan; 2 Ph.D. program for the Clinical Drug Discovery from Botanical Herbs, Taipei Medical University, Taipei, Taiwan; 3 Institute of Biomedical Sciences, Academia Sinica, Taipei, Taiwan; 4 Department of Aquaculture, National Kaohsiung Marine University, Kaohsiung, Taiwan; 5 School of Pharmacy, Taipei Medical University, Taipei, Taiwan; 6 Stemforce Biotechnology Co., Ltd, Chiayi City, Taiwan; 7 Graduate Institute of Medicine, Kaohsiung Medical University, Kaohsiung, Taiwan; 8 Graduate Institute of Pharmacognosy, Taipei Medical University, Taipei, Taiwan

## Abstract

The sensitivities of solid-phase immunoassays are limited by the quantity of detection antibodies bound to their antigens on the solid phase. Here, we developed a poly-protein G-expressing bacterium as an antibody-trapping microparticle to enhance the signals of immunoassays by increasing the accumulation of detection antibodies on the given antigen. Eight tandemly repeated fragment crystallisable (Fc) binding domains of protein G were stably expressed on the surface of *Escherichia coli* BL21 cells (termed BL21/8G). BL21/8G cells showed a higher avidity for trapping antibodies on their surface than monomeric protein G-expressing BL21 (BL21/1G) cells did. In the sandwich enzyme-linked immunosorbent assay (ELISA), simply mixing the detection antibody with BL21/8G provided a detection limit of 6 pg/mL for human interferon-α (IFN-α) and a limit of 30 pg/mL for polyethylene glycol (PEG)-conjugated IFN-α (Pegasys), which are better than that of the traditional ELISA (30 pg/mL for IFN-α and 100 pg/mL for Pegasys). Moreover, the sensitivity of the Western blot for low-abundance Pegasys (0.4 ng/well) was increased by 25 folds upon mixing of an anti-PEG antibody with BL21/8G cells. By simply being mixed with a detection antibody, the poly-protein G-expressing bacteria can provide a new method to sensitively detect low-abundance target molecules in solid-phase immunoassays.

## Introduction

Immunoassays of different formats, including enzyme-linked immunosorbent assay (ELISA) and Western blot, have been developed and widely applied to clinical diagnosis and pharmaceutical research^1–3^. Despite encompassing a broad range of techniques, the common principle of various immunoassays relies on the specific binding of detection antibodies to target molecules (antigens). The antigens are initially immobilised on a solid phase (a multi-well plate or a nitrocellulose membrane), so that they can interact with detection antibodies. However, a low number of antigens will only accumulate a low number of detection antibodies, which will produce a correspondingly small signal and this will therefore affect the sensitivity of the immunoassays. This physical limitation can cause antigens to become undetectable in the window period of diagnostic tests^2,4^. For example, p24 antigen, a biomarker for early HIV infection, is only detectable by ELISA 14 days after the initial infection^4^. Additionally, pharmacokinetic studies of protein drugs also involve measurements made by using specific antibodies^5,6^, and a highly sensitive immunoassay may be required to determine the concentration in a volume-limited sample^7^. Therefore, any means of improving the accumulation of detection antibodies would be helpful for detecting low-abundance targets.

Antibody-coated particles have been developed in order to increase the quantity of a detection antibody that interacts with a target molecule^8–10^. Owing to the high density of detection antibody on a nanoparticle, there will be substantial antibody-antigen interaction when detection antibody-coated nanoparticles are used to bind to antigens, resulting in signal amplification. To prepare antibody-modified particles, the amine terminals on an antibody can be covalently coupled with cyanogen bromide (CNBr)-activated^11,12^ or n-hydroxysuccinimide-activated particles^13^. Unlike conventional modifications, antibodies can adsorb on gold nanoparticles because of electrostatic and hydrophobic interactions^14,15^. However, the random orientation of antibodies on such particles impairs the desired specific antibody-antigen binding^16–18^. In order to achieve oriented antibody immobilization, bacterial immunoglobulin (Ig)-binding proteins such as protein A and protein G can be utilized to specifically interact with the fragment crystallisable (Fc) region of antibodies with high affinity^19^. Unidirectionally oriented antibodies attached by protein G have been shown to exhibit at least 3-fold higher antigen-binding capacity than randomly oriented antibodies^20,21^. Nevertheless, the preparation of protein G-coated particles requires laborious procedures, including the production and purification of recombinant protein G^22–24^, chemical conjugation, and the removal of uncoated protein G. Thus, their use can drastically raise the cost of an immunoassay.

**Figure 1:**
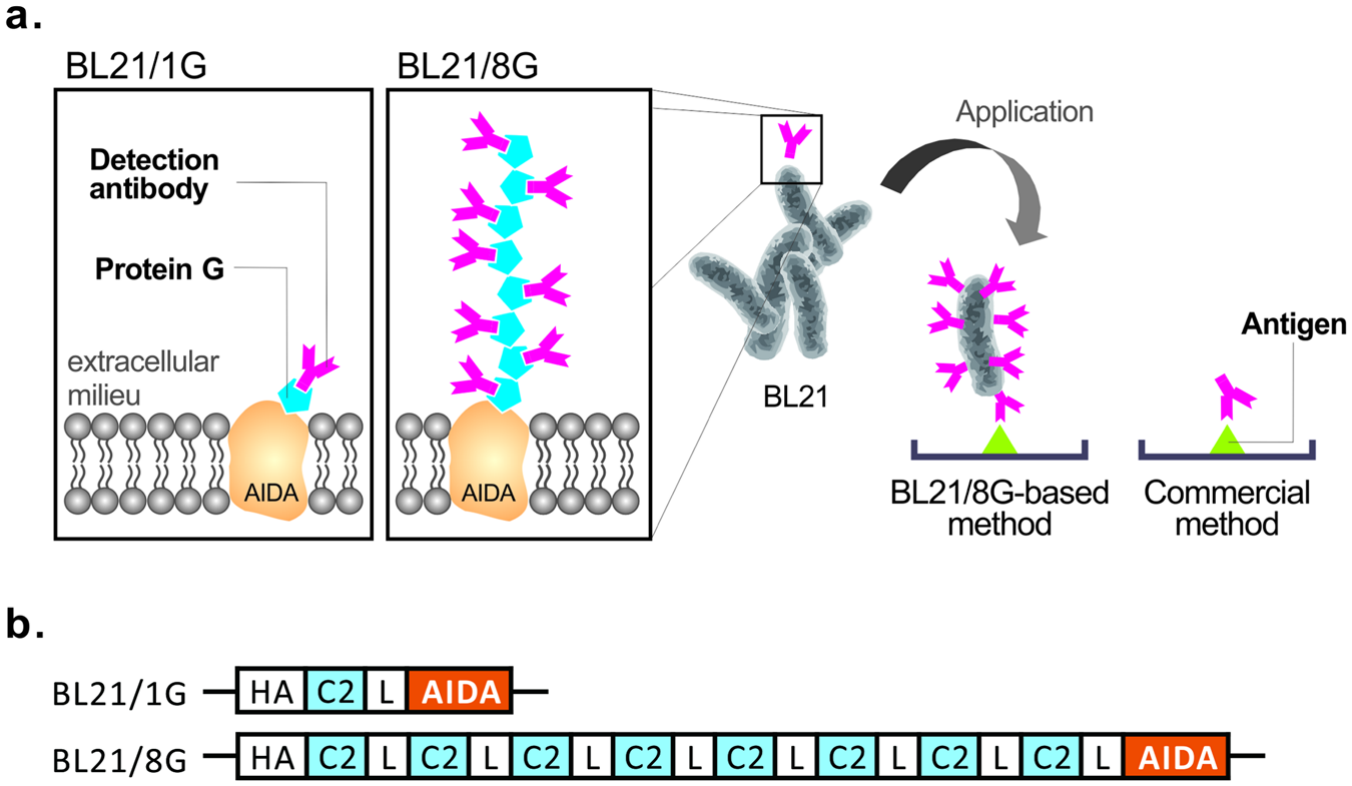
Poly-protein G-expressing BL21 bacteria. (**a**) Strategy for the non-covalent modification of detection antibodies to the single protein G C2 domain-expressing bacteria (BL21/1G) or the eight tandemly repeated C2 domain-expressing bacteria (BL21/8G). (**b**) Schematic representation of the gene construction of the protein G-expressing plasmids (1G and 8G). The construction includes, from N to C termini, an HA epitope tag, a single or eight tandemly repeated protein G C2 domain fragment (C2), a (GGGSG)3 linker (L), and an AIDA.

In this study, we describe a simple strategy for enhancing the sensitivity of immunoassays by using membrane-anchored protein G-expressing bacteria as a signal enhancer to improve the interaction of detection antibodies with target molecules. For this purpose, the C2 domain of streptococcal protein G, which has high specificity and affinity to the Fc domain of IgG antibodies^25–27^, was fused with the transmembrane domain of bacterial autotransporter adhesin involved in diffuse adherence (AIDA). The *Escherichia coli* BL21 cells stably expressed a single or eight tandemly repeated C2 domains on their cell surfaces, resulting in cells termed BL21/1G or BL21/8G cells, respectively. Compared to commercial immunoassays, those based on BL21/1G or BL21/8G cells allow more detection antibodies to interact with the antigen (Fig. 1a). These bacterial signal-enhancers can be mass-produced and can be easily conjugated with antibodies by a one-step mixing without purification. In this study, we compared the ability of BL21/1G cells and BL21/8G cells to trap detection antibodies by staining the cells with fluorescein isothiocyanate (FITC)- or horseradish peroxidase (HRP)-conjugated antibodies. To examine the signal enhancement yielded by BL21/1G and BL21/8G cells, we applied the cells in a direct ELISA by mixing the cells with an anti-polyethylene glycol (PEG) antibody (termed 6.3) to detect PEG molecules. We further tested whether the use of a mix of BL21/1G and BL21/8G cells would lower the detection limits for a human interferon-α (IFN-α) drug by anti-IFN-α antibody and for a PEG conjugated human IFN-α drug (Pegasys) by anti-PEG antibody in sandwich ELISA systems and Western blot.

## Results

### Surface display of a single or eight tandemly repeated protein G C2 domain on *Escherichia coli* BL21 cells

**Figure 2:**
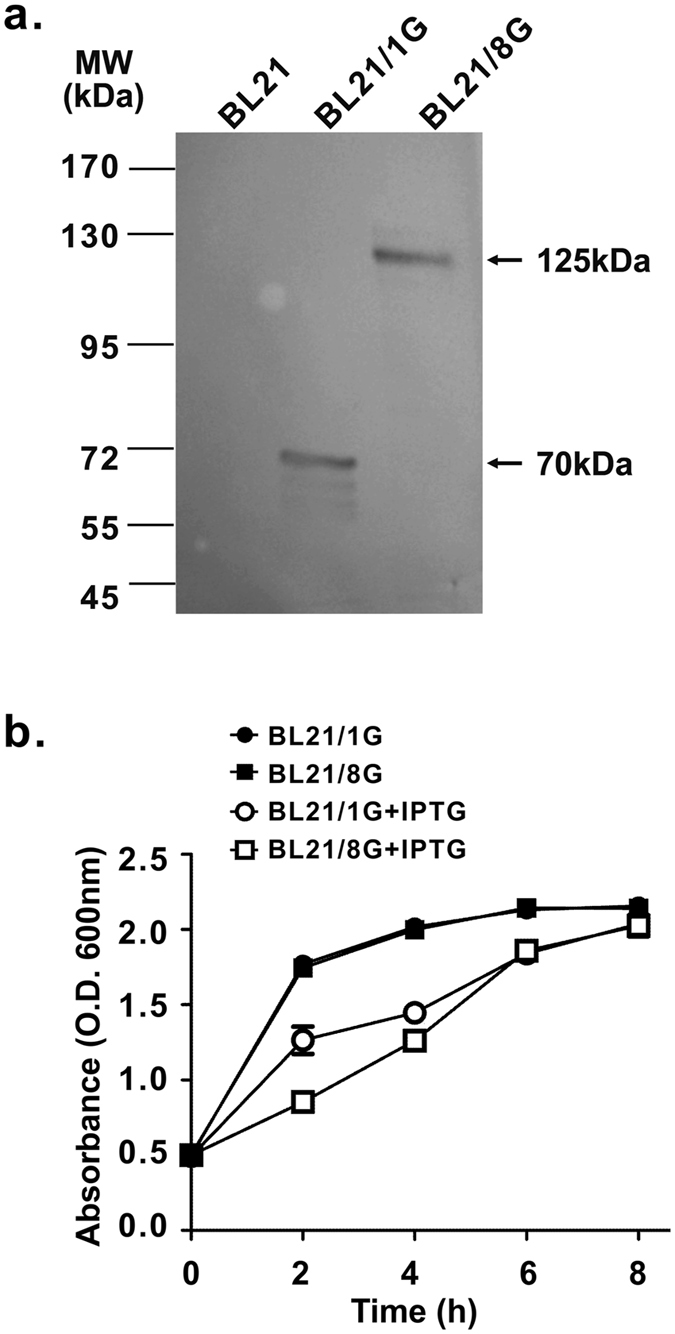
Expression of poly-protein G in BL21 bacteria. (**a**) The expression of poly-protein G in BL21 bacteria was analyzed by Western blot using a mouse anti-HA tag antibody. Lane 1, BL21 as negative control; Lane 2, single protein C2 domain-expressing BL21 bacteria (BL21/1G); Lane 3, eight tandemly repeated C2 domain-expressing BL21 bacteria (BL21/8G). (**b**) The transformed BL21 bacteria were induced by IPTG, and the growth rate of the bacteria was measured by recording the absorbance at 600 nm every two hours. (●)BL21/1G, (■)BL21/8G, (○)BL21/1G + IPTG, (□)BL21/8G + IPTG. Bar, SD.

The bacterial vectors pET22b-1G-AIDA and pET22b-8G-AIDA encode chimeric proteins in which a single or eight tandemly repeated protein G C2 domains were linked with AIDA (Fig. 1b). The pET22b-1G-AIDA and pET22b-8G-AIDA plasmids were transformed into *Escherichia coli* BL21 strains to generate the BL21/1G cells and BL21/8G cells, respectively. The expression of protein G receptors (1G-AIDA) or eight tandemly repeated protein G receptors (8G-AIDA) on the cellular surface was induced by isopropyl β-D-1-thiogalactopyranoside (IPTG). Western blot analysis showed that the bacteria did express 1G-AIDA and 8G-AIDA receptors with the expected sizes of 70 and 125 kDa, respectively (Fig. 2a). In addition, to investigate whether 1G-AIDA and 8G-AIDA receptors affect bacterial growth, bacteria density was monitored by measuring the optical density at 600 nm (O.D. 600 nm) every 2 h during IPTG induction. The expression of 1G-AIDA and 8G-AIDA receptors on the surface was slightly delayed but did not inhibit the growth rate of *Escherichia coli*. At the 8-hour time point, there was no significant difference in O.D. 600 nm between BL21/1G and BL21/1G with IPTG induction (p = 0.0584), or between BL21/8G and BL21/8G with IPTG induction (p = 0.0908) (Fig. 2b).

### The antibody-trapping ability of BL21/1G and BL21/8G

**Figure 3:**
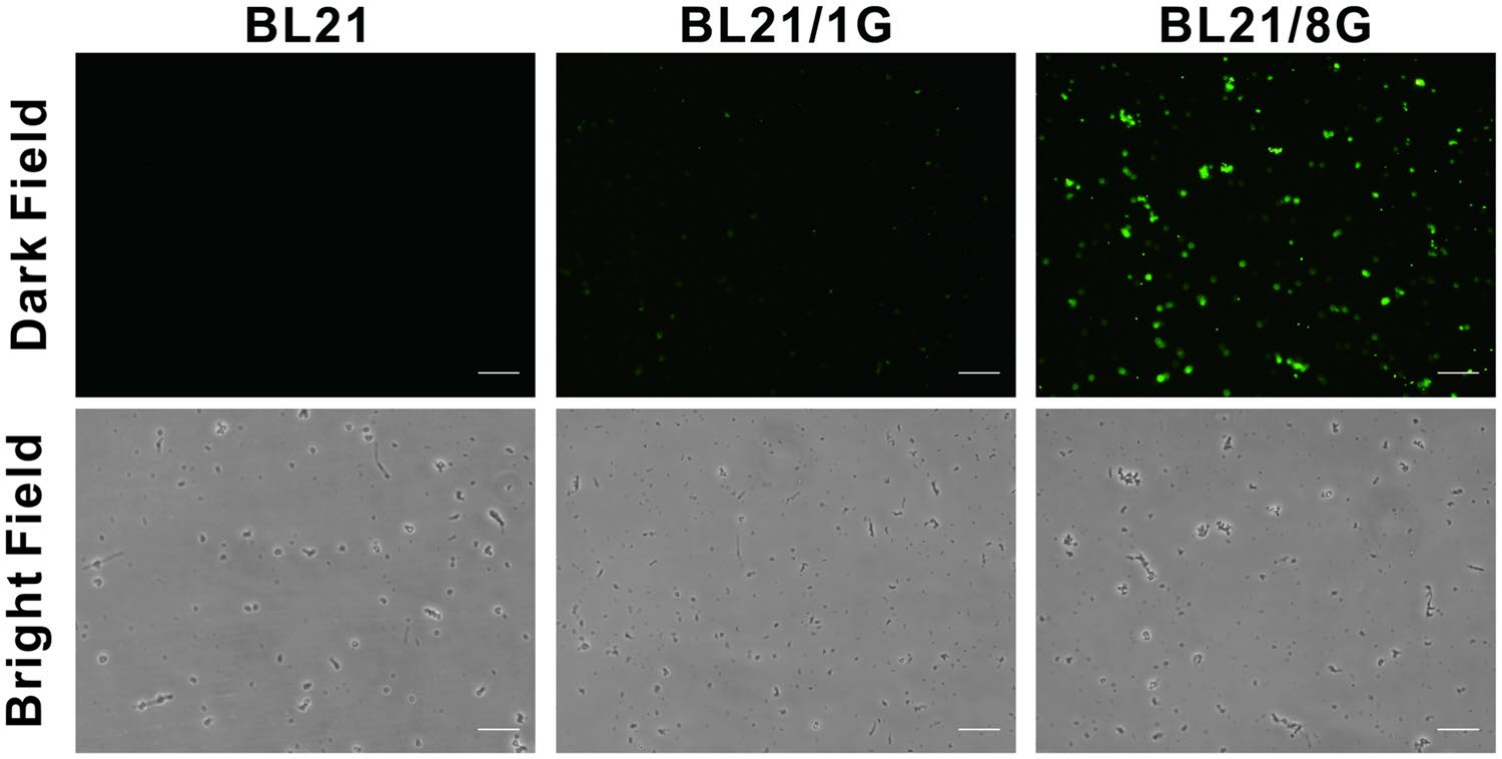
Fluorescent microscopy of poly-protein G-expressing bacteria labeled with FITC-conjugated antibody. The antibody-trapping ability of BL21, BL21/1G and BL21/8G was analyzed by FITC-conjugated goat antibody under a fluorescent microscope. Top panels, green fluorescence of FITC under dark field. Bottom panels, images of bacteria under bright field. Bar = 10 μm.

**Figure 4:**
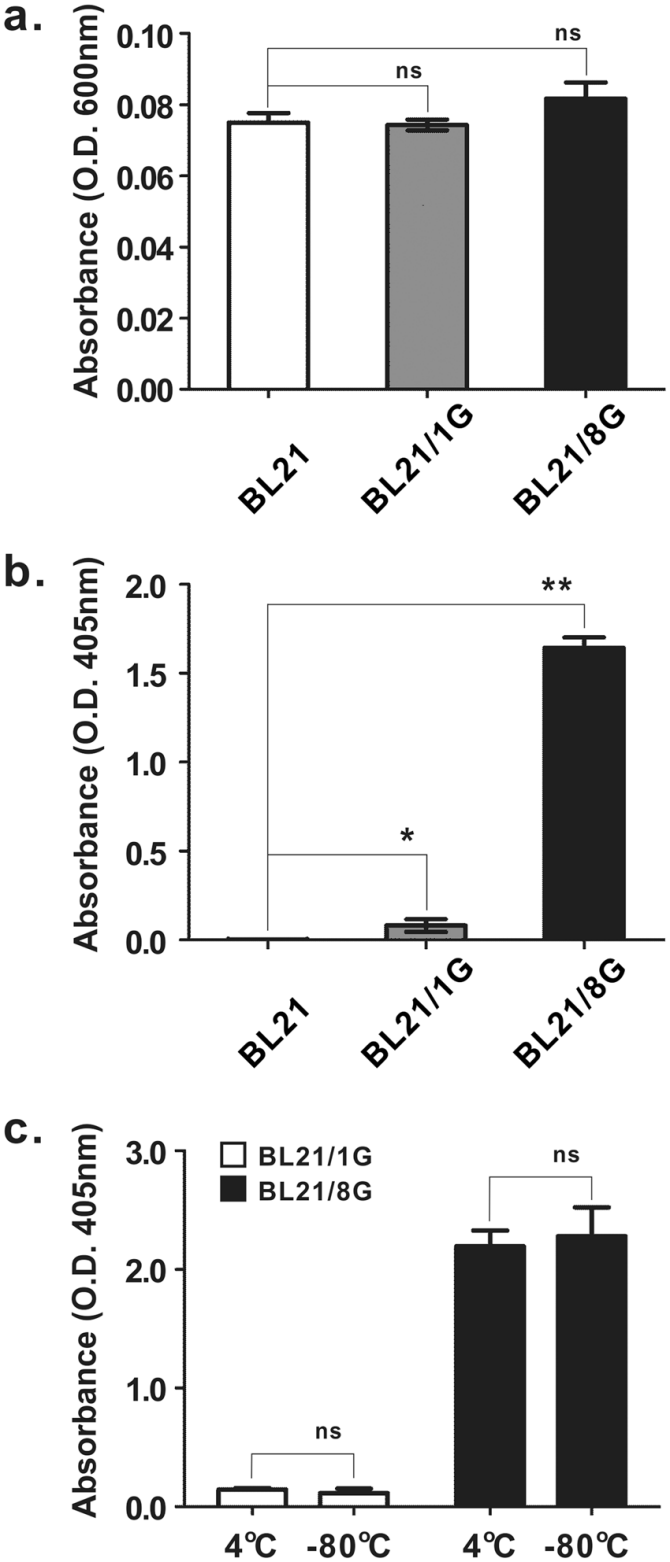
The antibody-trapping ability of poly-protein G-expressing bacteria. (**a**) Equivalent amounts of BL21, BL21/1G and BL21/8G cells were coated on 96-well plates. The absorbance of the bacteria was measured at 600 nm. (**b**) The antibody-trapping ability of BL21, BL21/1G and BL21/8G cells coated on 96-well plates was analyzed by HRP-conjugated goat antibody. Statistical analysis was performed by one-way ANOVA. (**c**) BL21/1G and BL21/8G cells were preserved in PBS containing 30% (v/v) glycerol at indicated temperatures for 40 days. The antibody-trapping ability of these bacteria was analyzed by HRP-conjugated goat antibody. Statistical analysis was performed by two-way ANOVA. Bar, SD. ns, no significant difference. *P value < 0.05; **P value < 0.001.

To assess the antibody-trapping ability of protein G-expressing bacteria, BL21, BL21/1G and BL21/8G cells were stained with FITC-conjugated goat antibody and then observed by fluorescence microscopy. Figure 3 shows that the fluorescent signal in the BL21/8G cells was obviously stronger than the signals in the BL21/1G and BL21 cells, indicating that the coating capacity of detection antibody on BL21/8G cells was much higher than those capacities for BL21/1G and BL21 cells. To further quantify the antibody-trapping effiency of protein G-expressing bacteria, BL21, BL21/1G and BL21/8G cells (about 6.7 × 10^6^ colony-forming units (cfu)/well) were coated in 96-well plates as determined by their optical density reading at 600 nm with a microplate reader (Fig. 4a). HRP-conjugated goat antibodies were added to these plates, respectively. The amount of the HRP-conjugated antibodies trapped on the cells was evaluated by the color development of 2,2′-azinobis (3-ethylbenzthiazoline-6-sulfonic acid) (ABTS) substrate. Figure 4b shows that the quantity of the antibody trapped on the BL21/8G cells was 18.9-fold higher than the quantity trapped on the BL21/1G cells, revealing that the antibody-trapping avidity of the 8G-AIDA receptor is significantly better than that of the 1G-AIDA receptor. In addition, Fig. 4c shows that storing the BL21/1G and BL21/8G cells at −80 °C with 30% (v/v) glycerol for 40 days did not harm their antibody-trapping ability, indicating that these bacterial cells could be preserved for long periods of time.

### One-step mixing of detection antibodies with BL21/8G cells enhances the detection signal of an ELISA system

**Figure 5:**
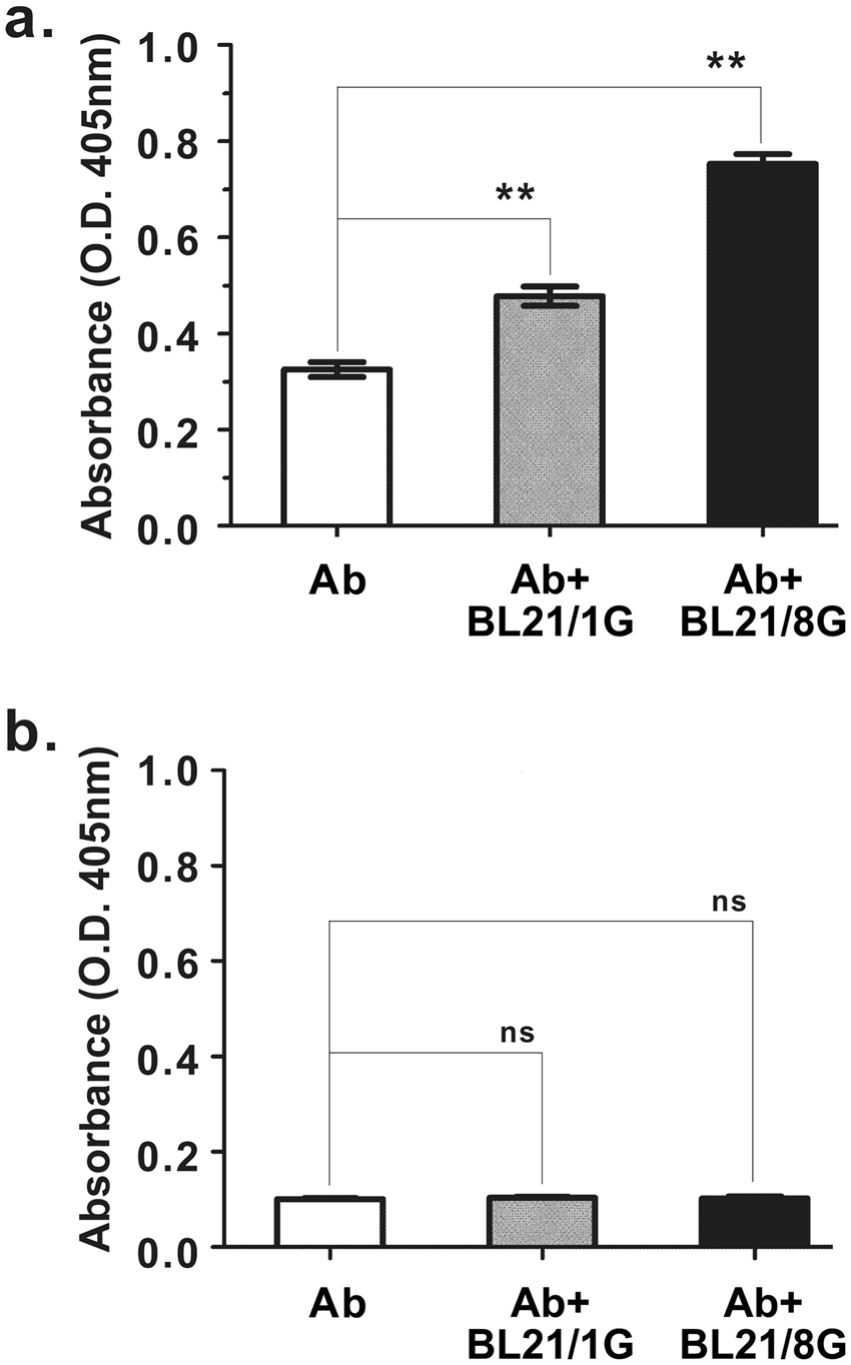
ELISA tests performed in the presence and absence of poly-protein G-expressing bacteria. Anti-PEG antibody (6.3-biotin) mixed with BL21/1G cells or BL21/8G cells, or not mixed with anything, was added into 96-well plates coated with (**a**) PEG_5k_-BSA or (**b**) BSA. The binding amount of 6.3-biotin was determined by measuring absorbance at 405 nm after staining with SA-HRP and ABTS. Ab, 6.3-biotin. Statistical analysis was performed by one-way ANOVA. Bar, SD. ns, no significant difference. **P value < 0.001.

To examine whether BL21/8G cells can enhance the detection signal of an ELISA system, biotin-conjugated anti-PEG antibody (6.3-biotin) was mixed with BL21/1G cells or BL21/8G cells, and then was added to 96-well plates coated with PEG (5 kDa)-modified bovine serum albumin (PEG_5k_-BSA) or bovine serum albumin (BSA). The binding amount of 6.3-biotin on a 96-well plate was detected by adding streptavidin-conjugated HRP (SA-HRP) and ABTS substrate. Figure 5a shows that the group of 6.3-biotin mixed with BL21/8G cells accumulated more 6.3-biotin on PEG_5k_-BSA than the other groups (6.3-biotin mixed with BL21/1G cells, or 6.3-biotin alone). None of these groups exhibited significant non-specific backgrounds in BSA-coated wells (Fig. 5b). These results demonstrated that one-step mixing of detection antibodies with BL21/8G cells can increase the accumulation of the antibodies on targeted molecules and, thus, can enhance the detection signal in an ELISA system.

### Development of a sensitive BL21/8G-based sandwich ELISA

**Figure 6:**
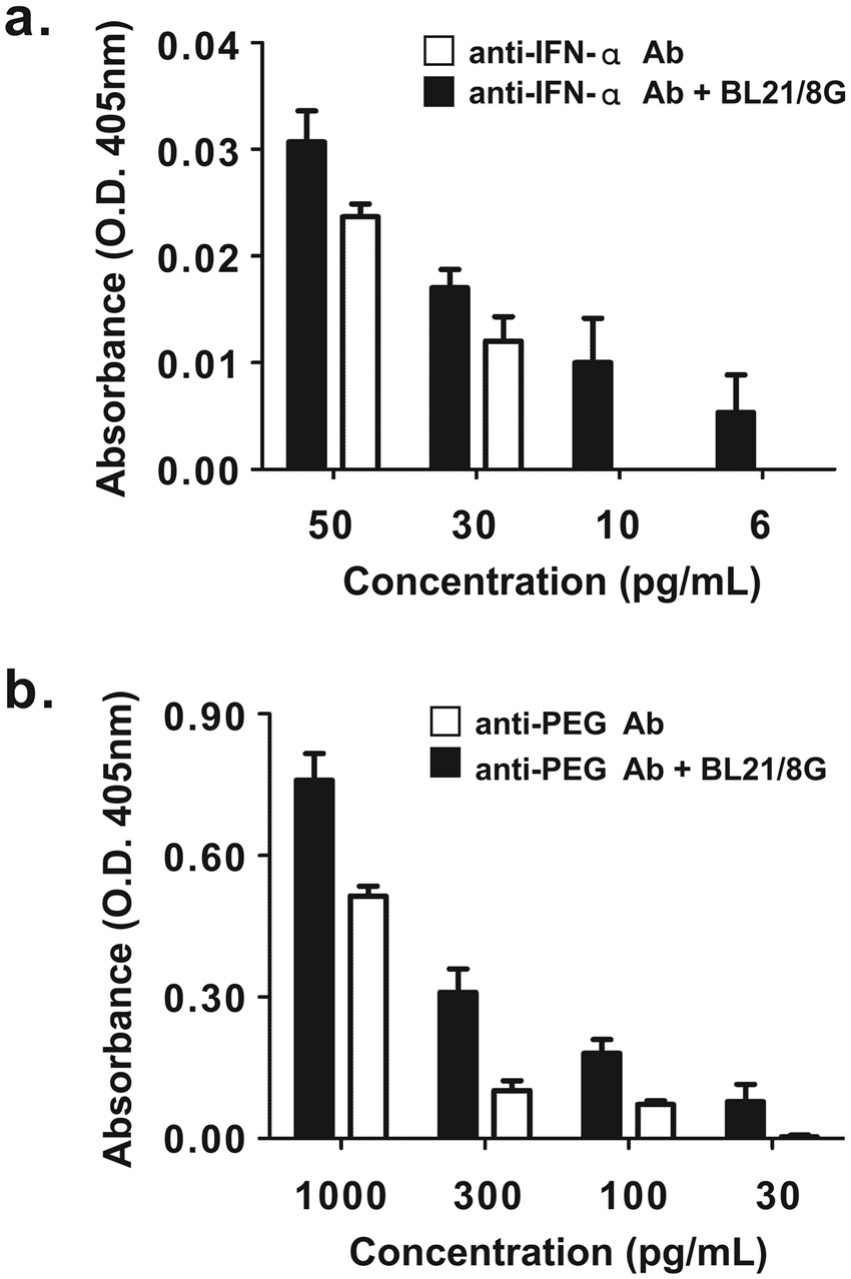
Detection of IFN-α and Pegasys by traditional sandwich ELISA or BL21/8G-based sandwich ELISA. (**a**) Serial diluted IFN-α samples were added into 96-well plates coated with anti-IFN-α capture antibody (MT1, IgM). Captured IFN-α molecules were detected by biotin-conjugated anti-IFN-α detection antibody (MT2, IgG) mixed with BL21/8G cells or not mixed with BL21/8G cells, and then color was developed using ABTS. (**b**) Serially diluted Pegasys samples were added into 96-well plates coated with anti-PEG capture antibody (AGP4, IgM). Captured Pegasys molecules were detected by biotin-conjugated anti-PEG detection antibody (3.3-biotin, IgG) mixed with BL21/8G cells or not mixed with BL21/8G cells, and color was developed using ABTS. Statistical analysis was performed by an independent t-test. Bar, SD.

We further examined whether BL21/8G cells can enhance the detection limit in two kinds of commercial sandwich ELISA kits, an anti-IFN-α sandwich ELISA kit and an anti-PEG sandwich ELISA kit, respectively. After the addition of defined concentrations of IFN-α to 96-well plates coated with an anti-IFN-α antibody (termed MT1), captured IFN-α molecules were detected by adding the detection antibody, a biotin-conjugated anti-IFN-α antibody (termed MT2), mixed with or without BL21/8G cells, followed by the addition of SA-HRP and ABTS substrate. Figure 6a shows that the BL21/8G group could sensitively detect IFN-α at concentrations as low as 6 pg/mL, whereas the detection limit of the commerical sandwich ELISA kit was 30 ng/mL. A similar result was also shown for an anti-PEG sandwich ELISA system using a IgM type anti-PEG antibody (termed AGP4) as the capture antibody and a biotin-conjugated IgG type anti-PEG antibody (termed 3.3-biotin) as the detection antibody for measuring Pegasys. The detection limit of the BL21/8G group (30 pg/mL) was superior to that of the commercial sandwich ELISA kit (100 pg/mL) (Fig. 6b).

### Development of a sensitive BL21/8G-based Western blot

**Figure 7:**
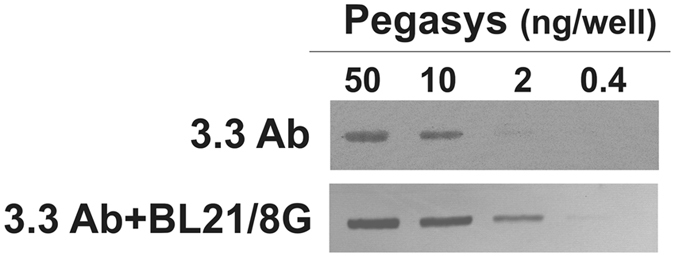
Western blot of PEG-conjugated IFN-α (Pegasys). Serially diluted Pegasys samples were electrophoresed on a 10% (w/v) reducing SDS-PAGE, transferred to nitrocellulose membrane, and probed with anti-PEG antibody (3.3) mixed with BL21/8G cells (lower panel) or not mixed with BL21/8G cells (upper panel) as described in the Materials and Methods section.

Insufficient binding of detection antibodies to low-abundance targeted molecules on an immunoblot membrane often limits the sensitivity of Western blot systems. We thus applied BL21/8G cells as a signal enhancer of an anti-PEG antibody (termed 3.3) to sensitively detect Pegasys in a Western blot system. Figure 7 shows that the detection limit of 3.3 for Pegasys was enhanced to 0.4 ng/well after it was mixed with BL21/8G cells, while the sensitivity with the conventional method was 10 ng/well. The application of BL21/8G cells can thus be said to increase the sensitivity of this anti-PEG antibody for low-abundance Pegasys by 25 times.

## Discussion

In this study, we present surface protein G-expressing bacteria (namely, BL21/1G and BL21/8G cells) as antibody-trapping microparticles that can be used to improve the detection sensitivities and limitations of immunoassays by increasing the accumulation of detection antibodies on a targeted antigen. The polymeric protein G-expressing bacteria (BL21/8G) showed a better capacity for trapping antibodies than did the monomeric protein G-expressing bacteria (BL21/1G) or BL21. The BL21/8G cells can be applied directly to different ELISA and Western blot systems to enhance their sensitivities through one-step mixing with the given detection antibody. Compared to conventional sandwich ELISA and Western blot, BL21/8G cells can enhance the sensitivity of detection antibodies by at least 3 folds in sandwich ELISA systems (Fig. 6) and by 25 folds in a Western blot (Fig. 7). Additionally, the BL21/8G cells are easily mass-produced and can be conjugated to any of the various detection antibodies through one-step mixing. The advantages of signal enhancement and one-step non-covalent modification using the BL21/8G cells indicate its potential value for application in various immunoassays.

The feasibility of the easy and rapid development of protein G-coated particles is very important if such products are to be widely accepted by customers. Conventional methods for developing protein G-coated particles with a consistent orientation include the production of the core particles, the production and purification of recombinant protein G, and the removal of unconjugated protein G. This complex procedure means that the resulting product is rather costly to produce. In addition, protein G-coated particles produced in this way cannot be frozen for storage, thus limiting their preservation. In contrast, the protein G-expressing bacteria (BL21/8G) produced for this study exhibited a number of advantages over conventionally produced protein G-coated particles. First, *Escherichia coli* has fast growth kinetics; its doubling time is about 20 minutes^28^ and the cell density of *Escherichia coli* in a culture bioreactor can reach over 100 g dry cell weight/L in simple glucose-salts media^29^. A large amount of BL21/8G cells can thus be easily obtained without additional purification, making the cost of producing BL21/8G cells much lower than the cost of producing traditional protein G-coated particles. Second, the display of protein G on the surface of BL21/8G cells is unidirectional (outward), allowing detection antibodies to be trapped on the BL21/8G cell surfaces with the same orientation, which can enhance the antigen-binding efficacy and detection sensitivity of antibodies in immunoassays^22,30^. Third, the BL21/8G cells can be stored at −80 °C with 30% (v/v) glycerol (Fig. 4c) or can be lyophilized and maintained in cryopreservation for more than 12 months^31,32^, which meets the required shelf life of commercial protein G-coated particles. These great characteristics of the BL21/8G cells would allow it to be easily adopted in general laboratories and industries.

The quantity of antibody trapped by BL21/8G cells was expected to be 8 folds higher than that trapped by BL21/1G cells. Nonetheless, Fig. 4b shows that the quantity trapped by the BL21/8G cells was far greater than just 8 times that trapped by the BL21/1G cells. There are two possible reasons for this phenomenon. First, a chain of eight tandemly repeated Fc-binding C2 domains is longer than a single C2 domain, and this longer poly-peptide has a larger swinging range, allowing it to efficiently trap antibodies in solution. Second, the binding avidity of a peptide drastically increases after it becomes a polypeptide. For example, Murata *et al*.^33^ showed that a polymeric arginylglycylaspartic acid (RGD) peptide competitively inhibited the binding of fibronectin to the cell surface more potently than an oligo RGD peptide, and that the polymeric RGD peptide was able to inhibit lung metastasis more effectively than the oligo RGD peptide^34^. In addition, Nagashima *et al*.^35^ developed an antibody with three tandemly repeated Fc domains at the c-terminus, and found that it exhibited about 100 times greater avidity than wild type antibody for Fc receptors (FcRIIA, FcRIIB and FcRIIIA). Therefore, the amount of antibody that the protein G C2 domain can trap appears to be dramatically increased after it is rendered into a repetitive polymer.

It seems that expressing longer polymeric C2 domains on the surface of bacteria allows antibodies to become more sensitive at detecting antigen in immunoassays. However, the over-expression of recombinant proteins, especially polymeric proteins, in host cells may affect their normal proliferation and homeostasis, resulting in slower growth rates, low final cell densities, and even the death of host cells^36,37^. It has been demonstrated, however, that using *Escherichia coli* to produce streptococcal protein G neither harms the immunoglobulin binding activity of protein G nor damages the *Escherichia coli*^38^; thus, *Escherichia coli* has become the major platform for the mass production of recombinant or truncated protein G^39^. In addition, we selected AIDA, a component of the bacteria beta-autotransporter system, as a transmembrane domain to stably anchor the protein G C2 domain on the bacterial surface. AIDA has been widely used to express a variety of proteins^40,41^ or enzymes^42^ on bacterial surfaces without affecting protein activity^43,44^. In this study, although both the BL21/1G and BL21/8G cells exhibited about a 2-fold slower growth rate after IPTG induction than the wild type BL21 cells, the final cell densities of the three strains were identical. Moreover, Fig. 2a also demonstrates that the expression levels of monomeric protein G (1G) and polymeric protein G (8G) in BL21 were similar, indicating that polymeric protein G can be stably and adequately expressed on the bacterial surface and that it does not harm the normal proliferation and homeostasis of the bacteria.

We demonstrated that the protein G-expressing bacteria BL21/8G can non-covalently conjugate to any of the various detection antibodies to increase their sensitivity in ELISA and Western blot. Moreover, the BL21/8G cells have a great potential to be applied to different kinds of antibody-related methodologies. For example, immunohistochemistry can identify the distribution of biomarkers and differentially expressed proteins in discrete tissue components by exploiting the interaction of target antigens with detection antibodies that have been tagged with an enzyme or a fluorescent dye^45,46^. The one-step mixing of these detection antibodies with BL21/8G cells before histological staining can increase the amount of detection antibodies at antigen sites, which we expect would in turn enhance the visualization of the antigens of interest in samples. Also, fluorescent dye-conjugated antibodies are widely used in flow cytometry and fluorescent microscopy to detect the expression of specific markers on cell surfaces^47,48^. The antibody-BL21/8G complex allows for greater accumulation of detection antibodies as well as fluorophores at surface marker sites, which could aid researchers in easily detecting specific markers with low expression levels on cells. Further experiments will be conducted to investigate the application of the BL21/8G cells in different antibody-related methodologies.

In this study, we developed a poly-protein G-expressing bacterial strain (BL21/8G) for use as an antibody-trapping microparticle in immunoassays. The strong antibody-binding capacity of the BL21/8G cells improved the detection sensitivity of ELISA and Western blot by increasing the amount of detection antibody accumulated on the given target antigen. Also, the antibody-trapping microparticle can be simply and rapidly generated via a one-step mixing of the BL21/8G cells and a detection antibody without the loss of antibody function. Moreover, the BL21/8G cells can be preserved at −80 °C without affecting its antibody-trapping ability. Based on these contributions, we believe that the BL21/8G cells may become a valuable tool for enhancing the capacities of various kinds of immunoassays to detect antigens in microbiology and oncology research, or to measure drug concentrations in pharmacokinetic studies.

## Materials and Methods

### Reagents and bacteria

*Escherichia coli* BL21 [F-ompT hsdSB (rB^−^, mB^−^) gal dcm (DE3)] was purchased from Millipore (Billerica, MA, USA). An IFN-α ELISA development kit was purchased from Mabtech (Victoria, Australia, product code: 3423-1H-6). Pegasys was purchased from Roche (Branchburg, NJ, USA). Two monoclonal mouse anti-PEG antibodies, termed 3.3 (IgG type) and AGP4 (IgM type), and two biotin-conjugated monoclonal mouse anti-PEG antibodies, termed 3.3-biotin (IgG type) and 6.3-biotin (IgG type), were provided by Dr. Steve R. Roffler (Academia Sinica, Taipei, Taiwan)^49^. PEG_5k_-BSA was synthesized as previously described^33^.

### Plasmid construction

The coding sequence of the protein C2 domain was amplified by a polymerase chain reaction (PCR). The hemagglutinin epitope tag and the restriction sites (SfiI, XhoI, SalI, and ClaI) were introduced using the following primers: 5SfiI-XhoI-protein G C2 domain (5′-tgctgcggcccagccggccctcgagacttacaaacttgttattaat-3′), 3(G4S)3-protein G C2 (5′tgatccaccgcctcctgatccaccgcctccttcagtaactgt-3′), 3ClaI-SalI-(G4S)3 (5′-ccatcgatgtcgactgatccaccgcctcctgatccaccgcctcctgatcc-3′. The PCR fragment was digested with SfiI and ClaI, and was then inserted into pLNCX vector (Clontech, Mountain View, CA, USA) to form pLNCX-1G. The eight tandemly repeated protein G C2 domain plasmids were constructed using the Tomas Kempe’s method^50^. Briefly, each of the pLNCX-1G plasmids was digested by BamHI + XhoI and BamHI + SalI. The fragments containing the protein G gene were isolated and fused by ligase to generate a pLNCX-2G plasmid. These processes were repeated twice to develop a pLNCX-8G plasmid. The 1G and 8G fragments were digested with SfiI and SalI, and then were inserted into pET22b-AIDA plasmid (Merck Millipore) to form pET22b-1G-AIDA and pET22b-8G-AIDA, respectively.

### Western blotting analysis of protein G-expressing bacteria

*Escherichia coli* BL21 cells were transformed using pET22b-1G-AIDA or pET22b-8G-AIDA to form BL21/1G cells and BL21/8G cells, respectively. The expression of 1G-AIDA and 8G-AIDA in BL21 cells was induced by 200 μM IPTG for 6 h, and then analyzed using a Western blotting protocol modified from a well-established protocol^49^. The transformed BL21 bacteria were washed by phosphate buffered saline (PBS) (pH 7.5) and boiled in reducing loading buffer at 100 °C for 10 min. The boiled bacteria (10^5^ cfu) were loaded in a 10% (w/v) sodium dodecyl sulfate polyacrylamide gel (SDS-PAGE) and electrophoresed under reducing conditions, and then transferred to a piece of nitrocellulose membrane (Millipore) in transfer buffer (50 mM NaCl, 2 mM EDTA, 0.5 mM 2-mercaptoethanol, 10 mM tris-HCl, pH 7.5). After being blocked in PBS containing 5% (w/v) skim milk, the membrane was incubated sequentially with 1 μg/mL mouse anti-hemagglutinin (anti-HA) antibody and 1 μg/mL HRP-conjugated goat anti-mouse IgG antibody (Jackson ImmunoResearch Laboratories, Westgrove, PA, USA) in PBS containing 2% (w/v) skim milk. The blots were washed with PBS containing 0.05% (v/v) Tween-20 (PBS-T) three times and with PBS twice before specific bands were visualized using electrochemiluminescent detection according to the manufacturer’s instructions (Pierce, Rockford, IL, USA).

### The growth rate of protein G-expressing bacteria

BL21/1G and BL21/8G cells were seeded in 30 mL of lysogeny broth medium containing 100 μg/mL of ampicillin. After reaching an O.D.600 nm of 0.5, the cells were either treated with 200 μM IPTG at 37 °C for 8 h or not treated with IPTG. The growth rate of these cells was measured by recording the absorbance at 600 nm using a MRX microplate reader (Dynex Technologies, Chantilly, VA, USA) every two hours. The statistical analysis was performed by an independent t-test.

### Analysis of antibody-trapping ability of protein G-expressing bacteria by fluorescent microscopy

BL21, BL21/1G, or BL21/8G cells (10^8^ cfu/500 μL) were added to a brown microcentrifuge tube and centrifuged at 6000G at 4 °C for 10 min. After discarding the supernatant, FITC-conjugated goat anti-mouse IgG antibody (1 μg/mL; MP Biomedicals, Santa Ana, CA, USA) was added and incubated on ice for 1 h. The bacteria were then washed three times with PBS containing 0.05% (w/v) BSA by centrifuge at 6000G at 4 °C for 10 min, and then were suspended with 50 μL of PBS containing 0.05% (w/v) BSA. The bacteria were then mixed 1:1 (v/v) with ProLong Diamond Antifade Mountant reagent (Thermo Fisher Scientific, Waltham, MA, USA) in a brown microcentrifuge tube. Next, 5 μL of the mixture was placed on coverslips then mounted on slides with nail polish. Images of the cells were captured using a fluorescence microscope (CKX41, Olympus, Tokyo, Japan) equipped with a 40x objective and a digital camera (E-330, Olympus).

### Analysis of antibody-trapping ability of protein G-expressing bacteria by ELISA

Maxisorp 96-well microplates (Nalge Nunc International, Rochester, NY, USA) were coated with poly-L-lysine in 50 μL/well of 0.1 M NaHCO_3_ (pH 8) at 37 °C for 1 h. BL21, BL21/1G and BL21/8G cells (10^7^ cfu/50 μL/well) in coating buffer (0.1 M NaHCO_3_, pH 9) were added and then were centrifuged at 3500 rpm at 4 °C for 30 min. After removing any uncoated bacteria by extensive washing, the plates were blocked overnight with 200 μL/well of 5% (w/v) skim milk at 37 °C for 2 h. The plates were next incubated in HRP-conjugated goat anti-mouse IgG Fc (1 μg/mL; Jackson ImmunoResearch) at room temperature for 1 h. The plates were then washed with PBS and the bound peroxidase activity was measured after adding 150 μL/well of ABTS substrate solution [0.4 mg/mL ABTS (Sigma-Aldrich, St. Louis, MO, USA), 0.003% (v/v) H_2_O_2_, and 100 mM phosphate-citrate, pH 4.0] at room temperature for 30 min. After color development, the absorbance was measured at 405 nm by a microplate reader.

### Preservation of BL21 in −80 °C

BL21/1G and BL21/8G cells were centrifuged at 3500 rpm at 4 °C for 1 h to remove the PBS supernatant. The bacteria were resuspended in PBS containing 30% (v/v) glycerol at the concentration of 10^10^ cfu/mL and then were preserved at −80 °C.

### BL21/8G-based ELISA

Maxisorp 96-well microplates were coated with PEG_5k_-BSA or BSA in 50 ng/50 μL/well of 0.1 M NaHCO_3_ (pH 9.0) at 37 °C for 2 h and then blocked with 200 μL/well of 5% (w/v) skim milk for 2 h at 37 °C. Biotin-conjugated anti-PEG antibody (6-3-biotin, 2 μg/mL) was mixed 1:1 with 4 × 10^8^ cfu/mL of BL21/1G cells or BL21/8G cells at 4 °C for 1 h (thus, the final concentration of 6-3-biotin was 1 μg/mL), and then the mixtures were added to microplates coated with PEG_5k_-BSA or BSA for 1 h at room temperature. After extensive washing, the microplates were sequentially incubated with SA-HRP and ABTS for color development. The absorbance was measured at 405 nm by a microplate reader.

### BL21/8G-based sandwich ELISA

An IFN-α ELISA development kit was used to conduct this experiment. Initially, 96-well microplates were coated with monoclonal anti-IFN-α antibody (MT1) in 100 ng/50 μL/well of PBS (pH 7.4) at 37 °C for 2 h and were then blocked with 200 μL/well of 3% (w/v) BSA at 37 °C for 2 h. IFN-α samples were serially diluted in PBS (pH 7.4) containing 2% (w/v) BSA and then were added to microplates for 2 h at room temperature. Biotin-conjugated monoclonal anti-IFN-α antibody (MT2) was mixed 1:1 with 4 × 10^8^ cfu/mL of BL21/8G cells for 1 h at 4 °C (thus, the final concentration of MT2 was 1 μg/mL), and then the mixtures were added to microplates at room temperature for 1 h. The microplates were sequentially incubated with SA-HRP and ABTS. Color development was measured at 405 nm by a microplate reader.

An anti-PEG sandwich ELISA kit using AGP4/3.3-biotin as the capture/detection pairing was used to measure Pegasys^51^. Initially, 96-well microplates were coated with AGP4 in 250 ng/50 μL/well of PBS (pH 7.4) at 37 °C for 2 h and were then blocked with 200 μL/well of 3% (w/v) BSA at 37 °C for 2 h. Pegasys samples were serially diluted in PBS (pH 7.4) containing 2% (w/v) BSA and then were added to microplates for 2 h at room temperature. 3.3-biotin was mixed 1:1 with 4 × 10^8^ cfu/mL of BL21/8G cells for 1 h at 4 °C (thus, the final concentration of 3.3-biotin was 1 μg/mL), and then the mixtures were added to microplates at room temperature for 1 h. The microplates were sequentially incubated with SA-HRP and ABTS. Color development was measured at 405 nm by a microplate reader.

### BL21/8G-based Western blotting analysis

Samples of Pegasys (50, 10, 2, and 0.4 ng) were electrophoresed in a 10% (w/v) SDS-PAGE under reducing conditions overnight before being transferred to a piece of nitrocellulose membrane by wet transfer in a blotting buffer (50 mM NaCl, 2 mM EDTA, 0.5 mM 2-mercaptoethanol, 10 mM tris-HCl, pH 7.5). Blots were blocked for 2 h with 5% (w/v) skim milk. Mouse anti-PEG antibody (3.3) (2 μg/mL) was mixed 1:1 with 4 × 10^8^ cfu/mL of BL21/8G cells at 4 °C for 1 h (thus, the final concentration of 3.3 was 1 μg/mL), and then were added to blots at room temperature for 1 h. The blots were washed with PBS-T three times and with PBS twice before incubation with HRP-conjugated goat anti-mouse antibody at room temperature for 1 h. The blots were then washed with PBS-T three times and with PBS twice before specific bands were visualized using an electrochemiluminescent substrate kit.

### ELISA data analysis

All the readings were background-adjusted by subtracting the absorbance of a blank control in the ELISA procedure. In Figs 4a,b and 5, the statistical differences between the groups were analyzed by one-way ANOVA. In Fig. 4c, the statistical difference in the protein G function of bacteria stored at 4 °C and −80 °C was analyzed by two-way ANOVA. In Fig. 6, the independent t-test was used to determine if there was any statistically significant difference in the detection limits estimated by the conventional sandwich ELISA and those estimated by the BL21/8G-based sandwich ELISA. Data were considered significant at p ≤ 0.05.
