# Author Correction: Poly-protein G-expressing bacteria enhance the sensitivity of immunoassays

**DOI:** 10.1038/s41598-018-22117-y

**Published:** 2018-03-06

**Authors:** Wen-Rui Hao, Michael Chen, Yi-Jou Chen, Yu-Cheng Su, Chiu-Min Cheng, Hsiang-Yin Hsueh, An-Pei Kao, Yuan-Chin Hsieh, Johny Chang, Ming-Yang Tseng, Kuo-Hsiang Chuang

**Affiliations:** 1 Division of Cardiovascular Medicine, Department of Internal Medicine, Shuang Ho Hospital, Taipei Medical University, New Taipei City, Taiwan; 2 Ph.D. program for the Clinical Drug Discovery from Botanical Herbs, Taipei Medical University, Taipei, Taiwan; 3 Institute of Biomedical Sciences, Academia Sinica, Taipei, Taiwan; 4 Department of Aquaculture, National Kaohsiung Marine University, Kaohsiung, Taiwan; 5 School of Pharmacy, Taipei Medical University, Taipei, Taiwan; 6 Stemforce Biotechnology Co., Ltd, Chiayi City, Taiwan; 7 Graduate Institute of Medicine, Kaohsiung Medical University, Kaohsiung, Taiwan; 8 Graduate Institute of Pharmacognosy, Taipei Medical University, Taipei, Taiwan

Correction to: *Scientific Reports* 10.1038/s41598-017-01022-w, published online 20 April 2017

The original version of this Article listed an incorrect Affiliation for Wen-Rui Hao. The correct Affiliation is listed below:

Division of Cardiovascular Medicine, Department of Internal Medicine, Shuang Ho Hospital, Taipei Medical University, New Taipei City, Taiwan.

This has now been corrected in the HTML and PDF versions of this Article.

